# Prevalence and stability of insufficient sleep measured by actigraphy: a prospective community study

**DOI:** 10.1038/s41390-020-0768-y

**Published:** 2020-01-31

**Authors:** Bror M. Ranum, Lars Wichstrøm, Ståle Pallesen, Silje Steinsbekk

**Affiliations:** 1 Department of Psychology, Norwegian University of Science and Technology, Trondheim, Norway; 2 NTNU Social Research, Trondheim, Norway; 3 Department of Child and Adolescent Psychiatry, St Olavs Hospital, Trondheim, Norway; 4 Department of Psychosocial Science, University of Bergen, Bergen, Norway

## Abstract

**Background:**

It is well established that reduced sleep has detrimental effects on school-aged children’s functioning, but the prevalence and stability of objectively measured insufficient sleep throughout childhood is unknown.

**Methods:**

A sample of 799 children was followed biennially with 24-h 7-day accelerometer (hip-placed) measurements from ages 6 to 12 years. Insufficient sleep was conceptualized as sleeping <7 h on average (AIS) and as the number of nights with <7 h of sleep (NNIS).

**Results:**

The prevalence of AIS ranged from 1.1% to 13.6%. Of those without AIS, 15.1–64.5% had >1 NNIS. At ages 6–10 years, NNIS was higher on weekend nights, but at age 12 years NNIS was lower on weekends (18.1%) compared to weekdays (23.4%). The stability of AIS was low from ages 6 to 8 years and from 8 to 10 years, but increased from age 10 to 12 years, whereas NNIS evidenced higher stability, increasing sharply through late middle childhood.

**Conclusions:**

The prevalence of AIS was low during the preschool and early school years but increased toward preadolescence. The 2-year stability of insufficient sleep was very low when conceptualized as AIS and moderate when defined as NNIS, hence NNIS might be more sensitive than AIS. Insufficient sleep appears transient in middle childhood and thus might not warrant intervention unless it fosters impairment and endures.

## Introduction

Insufficient sleep acutely impairs a range of human capacities, including cognition, emotion, and behavior regulation.^1^ There is no definite consensus as to what constitute insufficient sleep duration at different ages,^2^ as the optimal amount is challenging to pinpoint,^3^ and cultural variability in what is considered insufficient is substantial.^4^ Nevertheless, a substantial body of research has enabled researchers and clinicians to gain consensus on recommended sleep duration at different ages.^5^ For school-aged children (6–13 years), sleep duration <7 h is not recommended according to these guidelines (National Sleep Foundation (NSF)).^5^ Insufficient sleep duration is associated with concurrent physical and mental health problems,^6^ impaired school performance,^7^ and behavior problems^8^ in this age group. It is often measured by actigraphy, calculating a child’s average sleep duration over several days to determine whether it falls below a scientific consensus-based pre-defined cut-off (<7 h).^5^ Importantly, the prevalence and stability of objectively measured insufficient sleep throughout childhood is unknown. Moreover, by aggregating sleep assessments over several nights, it is also not known whether insufficient sleep mainly constitute weekday or weekend nights, although for the individual child this may make a difference. Perhaps even more importantly, one child might sleep quite well during most nights but have one nearly sleepless night, thus having a weekly average of <7 h. Another child may sleep less than recommended during all weekdays but “catch up” during weekends, thus bringing the average to >7 h. The first child would be considered to have insufficient sleep, whereas the second would be defined as being a normal sleeper. The first child might suffer the consequences of sleep deprivation for only the following day of his/her sleepless night, whereas the second child would have to cope with the adverse effects of sleep deprivation every single school day of the week and thus probably be more negatively affected than the first child. Critically, compensating for chronic short sleep duration by longer sleep on subsequent nights does not fully remediate the negative consequences of insufficient sleep^9^ because of an upregulation of adenosine receptor density, sensitizing the system and increasing sleep drive for a given number of consecutive hours. Thus, obtaining the recommended average does not necessarily constitute sufficient sleep if the child repeatedly has insufficient sleep during school days.

Acknowledging that sleep duration fluctuates across nights^10^ and that the negative outcomes of insufficient sleep may depend on this pattern of fluctuations, the present study aimed to investigate the prevalence of insufficient sleep conceptualized as the number of nights with insufficient sleep (NNIS) contrasted with averaged insufficient sleep (AIS) and to describe weekday–weekend differences.

Furthermore, if insufficient sleep persists, sleep debt may accumulate over extended periods, producing longer-term adverse effects,^9^ and interventions may thus be warranted. However, to decide whether or not to intervene, it is important to know whether insufficient sleep is typically a chronic or transient phenomenon in childhood; however, knowledge of the individual stability in objectively measured insufficient sleep is lacking. Sleep *duration* shows moderate-to-high stability over time in childhood (*r* = 0.32^11^ to *r* = 0.60^12^). However, stability in sleep duration does not translate well into stability of *insufficient sleep* (whether a child *consistently* gets insufficient sleep over time) for at least two reasons: first, a large majority of children sleep well.^13^ Hence, a correlation coefficient between sleep duration at two time points will be heavily influenced by those who sleep adequately at both time points. This may obscure changes from inadequate to adequate sleep. Second, a correlation coefficient is rank ordered. Hence, as the overall sleep duration by age declines among most children,^14^ some start to sleep less than the recommended duration, leading to an inflated measure of individual stability as measured by a correlation coefficient. Against this backdrop, we examined *individual stability* in objectively measured insufficient sleep on a night-to-night basis as well as on an average weekly basis, comparing the two different conceptualizations of prevalence and stability following a community sample of 6-year-olds with biennial assessments until the age of 12 years.

## Methods

### Participants and procedure

**Fig. 1:**
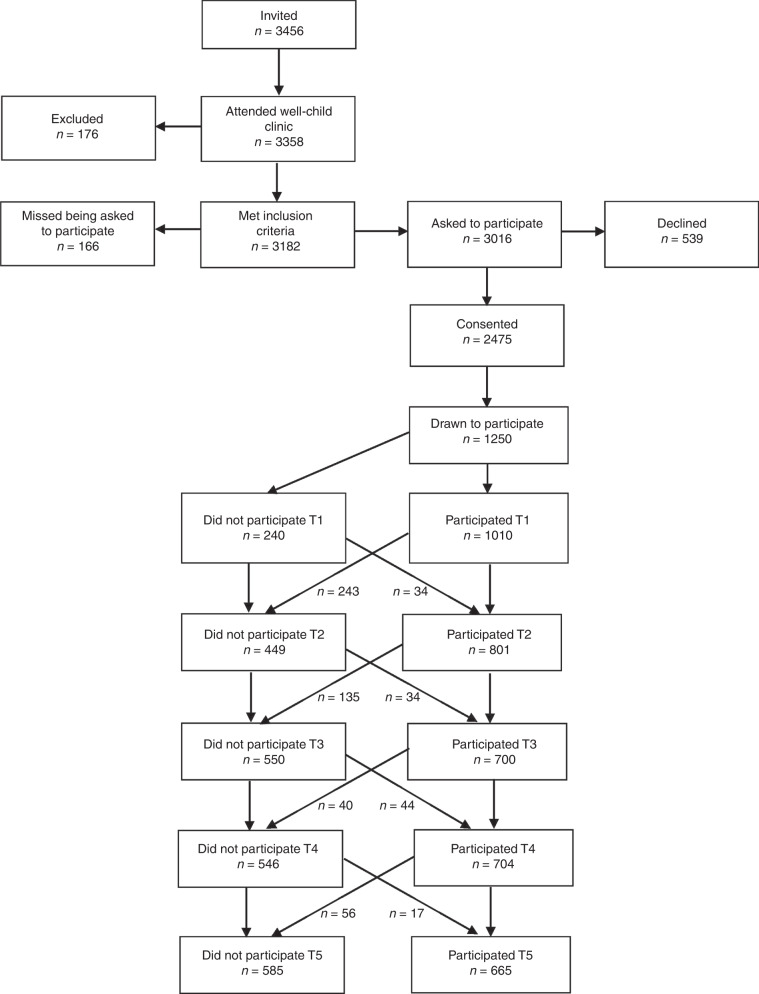
Sample recruitment. Number of participants at the various assessment points is based on the number of participants invited to participate (*n* = 1250) minus those who did not participate at the respective measurement point (i.e., T1, T2).

Data for the current study are derived from the Trondheim Early Secure Study (TESS).^15^ An invitation letter, together with a screen for emotional and behavioral problems (because the main aim of TESS was to study mental health), the Strengths and Difficulties Questionnaire (SDQ) 4–16 version,^16^ was sent to parents of all children in the 2003 and 2004 birth cohorts in Trondheim, Norway, together with their appointment for the age-4 routine health check-up (*N* = 3456). Almost all children appeared at the community clinic (*n* = 3358; 97.2%). The health nurse missed asking 166 parents, and 176 parents were excluded because lack of proficiency in Norwegian. The health nurse, using procedures approved by the Regional Committee for Medical and Health Research Ethics Mid-Norway, informed parents about TESS and collected written consents. A flowchart of the sample recruitment is presented in Fig. 1.

Children with emotional or behavioral problems were oversampled to increase statistical power; therefore, participants were allocated to four strata according to their SDQ scores (cut-offs: 0–4, 5–8, 9–11, and 12–40), and the probability of selection increased with increasing SDQ scores (0.37, 0.48, 0.70, and 0.89 in the four strata, respectively). We later applied population weights in our analyses to provide correct population estimates. Of the 1250 children randomly selected for the study, 997 were successfully enrolled (*M*_age_ = 4.7 years, SD = 0.3; 49.1% boys). The population of Trondheim was at the time of recruitment similar to the national average in Norway on several key indicators, i.e., employment rate, educational levels, and gross income.^17^ We compared the sample, adjusted for stratification, with register information from Statistics Norway on all parents of 4-year-olds in Trondheim in the years 2007 and 2008. The educational level was virtually identical to those of other parents in Trondheim. However, the sample contained more divorced parents (7.6%) than the general population (2.1%). The dropout rate after consenting to participate did not vary by SDQ score (*t* = 0.17, df = 1, *p* = 0.86) or gender (Cramer’s *v*^2^ = 1.02, df = 1, *p* = 0.31). Retesting occurred biennially at ages 6, 8, 10, and 12 years (*n* = 688, *M*_age_ = 6.0 years, SD = 0.17; *n* = 619; *M*_age_ = 8.8 years, SD = 0.24; *n* = 618, *M*_age_ = 10.5 years, SD = 0.17; *n* = 558, *M*_age_ = 12.5, SD = 0.14, respectively). Accelerometer assessment of sleep was conducted from age 6 years onwards; therefore, data from the first assessment were not included in the present report. Only participants with valid accelerometer data on at least one occasion were included (*n* = 799). Family situation (i.e., whether parents lived together or not), parent income, and child ethnicity did not predict inclusion to the analytic sample. Moreover, sleep duration, AIS, or NNIS predicted attrition on subsequent assessments. The sample characteristics are presented in Table 1.

**Table 1:** Sample characteristics of analytic sample at T2 (age 6 years).

Characteristic	Percentage
Gender of child	
Male	48.2
Female	51.8
Gender of parent informant	
Male	17.9
Female	82.1
Ethnic origin of biological mother	
Norwegian	93.5
Western countries	6.0
Other countries	0.5
Ethnic origin of biological father	
Norwegian	93.3
Western countries	6.0
Other countries	0.7
Biological parents’ marital status	
Married	59.4
Cohabitating	26.6
Divorced/separated	12.3
Other	1.7
Informant parents’ socioeconomic status	
Leaders	7.1
Higher professionals	27.4
Lower professionals	40.4
Skilled workers	22.4
Farmers/fishermen	0.2
Unskilled workers	2.5
Household gross annual income	
0–225’ NOK (0–26,500 USD)	2.2
225’–525’ NOK (26,500–62’ USD)	11.6
525’–900’ NOK (62’–106’ USD)	45.4
>900’ NOK (>106’ USD)	40.8

### Sleep measures

A triaxial ActiGraph GT3X accelerometer (Manufacturing Technology Incorporated, Fort Walton Beach, FL, USA) was used to assess children’s sleep. The accelerometer is a small device placed on the hip that measure acceleration in three dimensions. Children were instructed to wear the accelerometer for 7 consecutive days, 24 h a day, only taking it off when bathing or showering. Bedtime and rise time were manually set by inspecting each individual measurement. Researcher interpretations of bedtime and rise time are found to be closely aligned with sleep diary entries, with the resulting difference in sleep duration being miniscule (0.7 min).^18^ Sleep duration (minutes scored as sleep between bedtime and rise time, excluding minutes awake) was derived using the Sadeh sleep algorithm^19^ with 60-s epochs in the Actilife software (Actilife version 6.13.3. Actigraph, Pensacola, FL, USA). The Sadeh algorithm scores the current epoch as sleep or wake depending on the activity during the current epoch considering activity in the previous and subsequent 5 min, thereby also adjusting for night awakenings. Only nocturnal sleep was included. The data collected were used to define three sleep variables: (a) “AIS”—a dichotomization of average sleep duration <7 h vs. ≥7 h, with the cut-off set according to the NSF^5^; (b) “NNIS”; and (c) “weekend sleep”—a dichotomization of whether the night of insufficient sleep was followed by a weekend vs. weekday. Of note, the NSF recommends 9–11 h of sleep for children aged 6–13 years but also state that <7 h is “not recommended.” Because the aim of the present study is to capture prevalence and stability of insufficient sleep, we use the 7 h cut-off. Hip-placed accelerometers systematically overestimate sleep duration by approximately one 1 h,^20^ which was accounted for by subtracting 1 h from the accelerometer data prior to the analysis.

### Statistical analysis

All statistical analysis were performed in Mplus.^21^ Missing data were handled by applying full information maximum likelihood. Overall change at the population level in insufficient sleep throughout the observation period was assessed by the mean of a linear growth parameter in a latent growth model. To examine the possibility of different growth at different ages, piecewise growth curves were applied, comparing a model where the growths between two time points were set to be identical to a model where the growth was freely estimated, using the Satorra–Bentler scaled chi-square.^22^ To assess whether the individual stability of insufficient sleep differed across various age transitions, we compared an autoregressive model where stability coefficients were set to be equal between age periods with a model where coefficients were allowed to vary, using Satorra–Bentler scaled chi-square test. To assess whether nights with insufficient sleep were more prevalent on weekdays vs. weekends, we applied the parameter constraints test of means in Mplus, using Wald test. The stability of insufficient sleep was assessed with Pearson’s correlation (NNIS) and logistic regression (AIS).

## Results

### Prevalence

Very few 6-year-olds slept too little on average (AIS), but a slight increase was seen in middle childhood (ages 8 and 10 years), whereas at age 12 years, approximately 1 in every 7 children slept too little (Table 2). This seemingly linear increase was confirmed in a growth model (*M*_growth_ = 0.56, 95% confidence interval (CI), 0.21 to 0.92, *p* = 0.002, model fit: *χ*^2^ = 18.07, df = 10, *p* = 0.05) with no curvilinear effect (*M*_quadraticgrowth_ = -0.04, 95% CI, −0.20 to 0.11, *p* = 0.61, model fit: *χ*^2^ = 23.61, df = 6, *p* < 0.001). The number of nights with insufficient sleep was very low at age 6 years but increased throughout childhood (*M*_growth_ = 0.19; 95% CI, 0.16 to 0.21, *p* < 0.001, model fit: *χ*^2^ = 16.34, df = 5, *p* = 0.005, comparative fit index (CFI) = 0.781, Tucker–Lewis index (TLI) = 0.737, root mean square error of approximation (RMSEA) = 0.053 [90% CI: 0.026 to 0.083]). A quadratic solution had a poor fit to the data (*χ*^2^ = 11.98, df = 1, *p* < 0.001, CFI = 0.788, TLI = −0.273, RMSEA = 0.117 [90% CI: 0.064 to 0.181]). As Table 2 suggests, this may be due to a sharp increase from age 6 to 8 years, slightly leveling off to age 10 years, and then a sharp increase again—indicating a cubic-shaped development instead of a quadratic one. A cubic model cannot be estimated with four time points, but a piecewise growth curve analysis showed that the increase from age 6 to 8 years was indeed stronger than from age 8 to 10 years, Δ*χ*^2^ = 34.12, df = 1, *p* < 0.001, and the increase from age 10 to 12 years was also stronger than the increase from 8 to 10 years, Δ*χ*^2^ = 65.13, df = 1, *p* < 0.001. At age 12 years, children had on average two nights with insufficient sleep per week. Almost half of children had at least one night with insufficient sleep at ages 8 and 10 years, and at age 12 years, it was far more common than not to have at least one night of insufficient sleep.

**Table 2:** Averaged sleep duration, prevalence of averaged insufficient sleep and number of nights with insufficient sleep.

	Age 6 years	Age 8 years	Age 10 years	Age 12 years
Sleep duration (minutes), mean (SD)	579 (35)	550 (36)	539 (35)	519 (38)
Averaged insufficient sleep, % (95% CI)	1.1 (0.2–2.0)	3.9 (2.0–5.9)	4.2 (2.4–6.0)	13.6 (10.2–17.0)
Averaged *sufficient sleep* with one or more nights of insufficient sleep, % (95% CI)	15.1 (12.0–18.2)	39.1 (34.6–43.7)	45.7 (41.1–50.3)	64.5 (59.6–69.4)
Number of nights with insufficient sleep, no. (SD)	0.25 (0.67)	0.66 (0.98)	0.83 (1.13)	1.56 (1.53)

**Table 3:** Correlation between study variables.

	1	2	3	4	5	6	7	8	9	10	11
(1) Age 6 Averaged sleep duration											
(2) Age 8 Averaged sleep duration	0.33**										
(3) Age 10 Averaged sleep duration	0.41**	0.35**									
(4) Age 12 Averaged sleep duration	0.32**	0.32**	0.41**								
(5) Age 6 Averaged insufficient sleep	−0.40**	−0.15**	−0.17*	−0.16							
(6) Age 8 Averaged insufficient sleep	−0.09	−0.51**	−0.07	−0.00	0.08						
(7) Age 10 Averaged insufficient sleep	−0.17	−0.15*	−0.43**	−0.22**	0.28	0.01					
(8) Age 12 Averaged insufficient sleep	−0.30*	−0.22**	−0.28**	−0.66**	0.11	−0.01	0.18*				
(9) Age 6 Number of nights with insufficient sleep	−0.58**	−0.10	−0.19**	−0.21*	0.56**	0.03	0.26	0.21*			
(10) Age 8 Number of nights with insufficient sleep	−0.13*	−0.68**	−0.17**	−0.22**	0.08	0.53**	0.06	0.12*	0.04		
(11) Age 10 Number of nights with insufficient sleep	−0.28**	−0.29**	−0.70**	−0.37**	0.19*	0.03	0.53**	0.29**	0.21*	0.22**	
(12) Age 12 Number of nights with insufficient sleep	−0.32**	−0.29**	−0.38**	−0.77**	0.08	0.01	0.17*	0.64**	0.19*	0.23**	0.30**

*p < 0.05, **p < 0.01, ***p < 0.001.

At age 6 years, the nights with <7 h sleep were almost twice as prevalent on weekend nights (5.0%) compared to nights followed by a weekday (2.9%, Δ*χ*^2^ = 7.32, df = 1, *p* = 0.007). The same was true at ages 8 and 10 years, with the percentages of weekend nights being 14.2% and 16.4%, respectively; for weekday nights, they were 6.4% and 9.7%, respectively (Δ*χ*^2^ = 41.26, df = 1, *p* < 0.001 and Δ*χ*^2^ = 19.93, df = 1, *p* < 0.001). At age 12 years, this trend was reversed, as nights with <7 h of sleep were more prevalent on nights followed by a weekday (23.4%) than on weekend nights (18.1%, Δ*χ*^2^ = 6.03, df = 1, *p* = 0.014).

### Stability

**Fig. 2:**
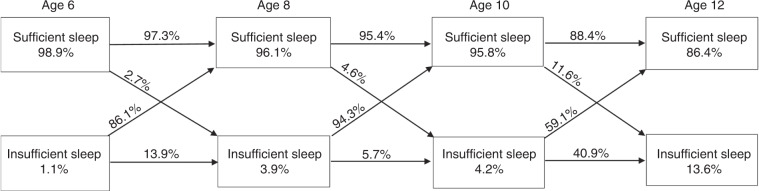
Flowchart of children moving between averaged sufficient and insufficient sleep.

AIS showed stability neither from age 6 to 8 years (odds ratio (OR) = 5.8, 95% CI, 0.59–57.32) nor from age 8 to 10 years (OR = 1.26, 95% CI, 0.15–10.46). However, some stability emerged from age 10 to 12 years (OR = 5.30, 95% CI, 1.77–15.82); 40% of 10-year-olds with AIS also had AIS at age 12 years (Fig. 2). Of note, the 10–12-year-old stability was significantly stronger than that of the 6–8- and 8–10-year transitions (Δ*χ*^2^ = 15.66, df = 1, *p* < 0.001). The 6–8- and 8–10-year stability was not significantly different from each other (Δ*χ*^2^ = 0.97, df = 1, *p* = 0.32). When insufficient sleep was conceptualized as NNIS, the rank order stability from age 8 to 10 years and from 10 to 12 years emerged as modestly to moderately stable (Table 2). Correlation between study variables is provided in Table 3.

## Discussion

The aim of the present study was, for the first time, to detail the stability of objectively measured insufficient sleep during childhood as well as to provide prevalence estimates when insufficient sleep was conceptualized as sleeping <7 h per night, on average, as opposed to the NNIS per week. Insufficient sleep, measured both as AIS and NNIS, was infrequent at age 6 years. At ages 8 and 10 years, the prevalence of AIS was low, whereas that of NNIS rose sharply. The latter increase became even more pronounced at age 12 years, with a prevalence of 14% for AIS and with 69% having at least one night with insufficient sleep. Thus an increasing and substantial proportion of children classified as having *sufficient* sleep on average had one or more nights of *insufficient* sleep. In middle childhood, nights with insufficient sleep were more prevalent on weekends than on weekdays, but in preadolescence, they were more prevalent on weekdays. In terms of the stability of sleep, AIS showed no 2-year stability at younger ages, but some stability emerged from age 10 to 12 years. In contrast, NNIS values were already moderately stable from age 8 years. Overall, NNIS emerged as a more sensitive measure of too little sleep than weekly averaged sleep duration.

### Prevalence of insufficient sleep

To our knowledge, no one has previously provided prevalence estimates of insufficient sleep for school-aged children. Nevertheless, comparing sleep durations for children in different countries may shed some light on the variability of insufficient sleep depending on the region. Although children’s sleep duration is found to vary depending on the region in survey studies,^23^ it does not vary in actigraphy studies.^14^ Therefore, we would expect to see similar prevalence rates of insufficient sleep in other countries; nevertheless, this remains to be investigated.

Regardless of conceptualization, insufficient sleep increased sharply with age in our study, which is consistent with studies showing a steady age decline in sleep duration during childhood and adolescence.^24^ The most apparent and simple explanation for this increase is that the need for sleep declines with age, whereas recommendations remain fixed for this age group. Another reason for this development may be that, with increasing age, parents more often leave decisions regarding bedtime to the child.^25^ At the age of 12 years, several children approach puberty, which is associated with a biologically based delayed circadian rhythm^26^ and a slowing of the homeostatic sleep pressure build-up.^26^ This, coupled with a stable school start time,^27^ often result in curtailed sleep. Moreover, extensive media use is associated with reduced sleep duration and later bedtimes^28^; the increase in insufficient sleep might therefore also be influenced by the increase in media use in preadolescence.^29^

Our finding of higher prevalence of nights with insufficient sleep on weekends than on weekdays during early middle childhood agrees with findings of shorter parent-reported sleep duration on weekdays for preschoolers and early school-aged children,^30^ but it does not agree with the results from a recent meta-analysis of objectively measured sleep that found no significant differences in sleep duration between weekend and weekday sleep at ages 9–11 years.^14^ Of note, unaltered averaged sleep duration might conceal increases in both longer and shorter sleep. Furthermore, as the weekday–weekend ratio was reversed after age 10 years, with preadolescents often sleeping too little during weekdays and “catching up” during weekends, the reason for the null finding in the meta-analysis may be that the 9–11-year age group includes both 9-year-olds sleeping less on weekends and 11-year-olds sleeping less on weekdays, thus canceling out the difference. Our results imply that the negative effects of too little sleep on school performance^7^ might—at the population level—have its origins somewhere between the ages of 10 and 12 years.

### Continuity of insufficient sleep

Our findings of objectively measured insufficient sleep having no stability during the first school years and only being modestly stable in middle childhood and preadolescence do not agree with previously reported findings of high stability for subjective reports of sleep problems^31^ and insomnia^32^ within the preschool and school-age periods, continuing into adolescence.^33^ Bearing in mind the modest concordance between parent reports and objectively defined sleep,^34^ one possible reason for the discrepancy between our and previous findings is that subjective proxy reports of children’s sleep tap into the stability of the rater’s perception, not necessarily stability in the child’s sleep.

As our results reveal, a child’s *averaged* insufficient sleep is more likely to pass than to persevere over a 2-year period. Hence, from this result alone, interventions targeting sleep duration appear to be needed less often than is commonly presumed.^35^ Nevertheless, a larger portion of children with AIS showed stability in having one or several nights with insufficient sleep. As a consequence of using the average, these bouts of insufficient sleep could pass unnoticed, as would important insights into the development, causes, and consequences of insufficient sleep in children. Even modest differences in sleep duration over only a few nights have been found to have significant consequences for children’s daytime functioning,^36^ implying that these bouts of insufficient sleep might be important. Whether AIS or NNIS has a greater impact on children’s functioning remains to be investigated.

### Strengths and limitations

The strengths of this study are the large and representative sample, the longitudinal design, and the objective sleep measurement over 7 days. We measured sleep using hip-placed accelerometers. Hip-placed measures have not been validated against polysomnography (PSG; the gold standard for evaluating sleep) for children aged 6–12 years; however, two studies have compared hip-placed with wrist-worn devices, reporting systematic differences of 7^18^ and 74 min,^37^ respectively, with correlations of *r* = 0.93 and *r* = 0.70, respectively. Moreover, for children aged 6–12 years, the accuracy of the wrist-worn accelerometer compared to PSG is high, ranging from *r* = 0.84^38^ to *r* = 0.90.^39^ Hence, from these results, we should expect hip-placed accelerometer data, when adjusted for overestimation, to compare well with PSG data. Such a conclusion is also supported by the fact that the sleep duration data in the current study were similar to those reported in a systematic review of observational studies on normal sleep patterns^23^ and to established normal values for pediatric nighttime sleep measured by actigraphy.^14^

Sleep duration typically declines with age within the childhood years,^40^ and it is reasonable to assume that this is due to a decline in sleep *need*. We applied the recommendations from the NSF, which is similar across the age 6–12-year range. In effect, the appropriateness of this cut-off may vary with age. Even so, because of the difficulty in pinpointing the exact optimal amount of sleep at different ages,^3^ to ease comparison with other research, as well to not risk “over-diagnosing” insufficient sleep, we chose to follow these widely used and rather restrictive guidelines.

Children with insufficient sleep at night may compensate by napping at daytime, thereby reaching the recommended amount. We did not include daytime sleep, as daytime naps are uncommon for school-aged children.

As children’s sleep duration data measured by actigraphy are found to be similar in different regions,^14^ our results may be generalized to other western industrialized countries. Given the large cultural variability in sleep behavior and interpretation of what is considered insufficient or problematic sleep^4^ the <7 h cut-off applied here—and hence the results—might not generalize to other cultures.

## Conclusion

Sleeping too little, i.e., <7 h, is rare among 6-year-olds, but it becomes slightly more prevalent in middle childhood and even more common in preadolescence. In middle childhood, insufficient sleep is twice as prevalent during weekends as it is on weekdays, but by preadolescence, the opposite pattern is observed: on average, every fourth night before a school day is a night with insufficient sleep. The 2-year stability of insufficient sleep is low in early middle childhood, but it increases thereafter: at age 10 years, 40% of cases with insufficient sleep persevere at age 12 years. Conceptualizing insufficient sleep as average sleep duration of <7 h across the week revealed many nights with insufficient sleep, and the stability of insufficient sleep was higher when measured as NNIS than as AIS. Hence, NNIS might be a more sensitive measure of insufficient sleep than reduced overall weekly sleep duration.
